# Climate Influences the Content and Chemical Composition of Foliar Tannins in Green and Senesced Tissues of *Quercus rubra*

**DOI:** 10.3389/fpls.2017.00423

**Published:** 2017-05-16

**Authors:** Sara M. Top, Caroline M. Preston, Jeffrey S. Dukes, Nishanth Tharayil

**Affiliations:** ^1^Plant and Environmental Sciences, Clemson UniversityClemson, SC, USA; ^2^Natural Resources Canada, Pacific Forestry CentreVictoria, BC, Canada; ^3^Department of Forestry and Natural Resources, Purdue UniversityWest Lafayette, IN, USA; ^4^Department of Biological Sciences, Purdue UniversityWest Lafayette, IN, USA; ^5^Department of Biology, University of Massachusetts BostonBoston, MA, USA

**Keywords:** hydrolysable tannins, condensed tannins, drought, warming, Proanthocyanidins, *Quercus rubra*

## Abstract

Environmental stresses not only influence production of plant metabolites but could also modify their resorption during leaf senescence. The production-resorption dynamics of polyphenolic tannins, a class of defense compound whose ecological role extends beyond tissue senescence, could amplify the influence of climate on ecosystem processes. We studied the quantity, chemical composition, and tissue-association of tannins in green and freshly-senesced leaves of *Quercus rubra* exposed to different temperature (*Warming* and *No Warming*) and precipitation treatments (*Dry, Ambient, Wet*) at the Boston-Area Climate Experiment (BACE) in Massachusetts, USA. Climate influenced not only the quantity of tannins, but also their molecular composition and cell-wall associations. Irrespective of climatic treatments, tannin composition in *Q. rubra* was dominated by condensed tannins (CTs, proanthocyanidins). When exposed to *Dry* and Ambient^*^Warm conditions, *Q. rubra* produced higher quantities of tannins that were less polymerized. In contrast, under favorable conditions (*Wet*), tannins were produced in lower quantities, but the CTs were more polymerized. Further, even as the overall tissue tannin content declined, the content of hydrolysable tannins (HTs) increased under *Wet* treatments. The molecular composition of tannins influenced their content in senesced litter. Compared to the green leaves, the content of HTs decreased in senesced leaves across treatments, whereas the CT content was similar between green and senesced leaves in *Wet* treatments that produced more polymerized tannins. The content of total tannins in senesced leaves was higher in *Warming* treatments under both *dry* and *ambient* precipitation treatments. Our results suggest that, though climate directly influenced the production of tannins in green tissues (and similar patterns were observed in the senesced tissue), the influence of climate on tannin content of senesced tissue was partly mediated by the effect on the chemical composition of tannins. These different climatic impacts on leaves over the course of a growing season may alter forest dynamics, not only in decomposition and nutrient cycling dynamics, but also in herbivory dynamics.

## Introduction

Global changes, through their widespread influence on all biological processes, can significantly impact plant life cycles and functions (Norby and Luo, 2004). Elevated levels of CO_2_ and ozone, increased temperatures, as well as higher drought frequency, all alter the content and composition of plant metabolites by disrupting the physiological stoichiometry in plants (Kuokkanen et al., 2001; Xu and Zhou, 2006; Prior et al., 2011; Xu et al., 2014, 2015), thus influencing plant survival and distribution. Furthermore, by influencing the chemical composition of senesced litter that fuels the metabolism of soil heterotrophs, the climate-induced physiological alterations in plants could also influence soil nutrient cycling and ecosystem productivity. For example, climate-induced changes in the composition of senescing leaves (Top and Filley, 2014; Suseela et al., 2015) could affect the subsequent detritivory (Currano et al., 2008; Couture et al., 2012) that regulates the cycling of soil carbon and mineral nutrients (Aerts, 1997; Liu et al., 2009; Suseela et al., 2013), which in turn could influence ecosystem productivity. These protracted influences of environmental stress on ecosystem performance could be mediated in part by climatic regulation of defense compounds that retain their biological-inhibitory properties even after tissue senescence.

Polyphenols, specifically tannins, are ecologically relevant, multifaceted carbon-based secondary metabolites that are common in most plant species (Kraus et al., 2003a). These compounds perform multiple protective functions and facilitate the interactions of plants with their biotic and abiotic environments during the active growth of tissues and after tissue senescence. In green tissues, as protective compounds, tannins play a critical role in plant—herbivore and plant-to-plant interactions (Salminen and Lempa, 2002; Kraus et al., 2003a; Barbehenn and Constabel, 2011), and also serve as a photo-protectant (Close and Mcarthur, 2002). Tannins in senescent leaves can account for up to 25% leaf dry weight (Kraus et al., 2003b) and thus can be a major form of carbon reaching the belowground ecosystems. Tannins in senescent tissues also retain their ability to complex with proteins (Hagerman et al., 1998) and inactivate soil enzymes (Triebwasser et al., 2012), which could hinder soil N mineralization (Hättenschwiler and Vitousek, 2000; Kraus et al., 2003a; Adamczyk et al., 2013; Tharayil et al., 2013). Thus, environmental conditions that exist during plant growth, by regulating the production of tannins in green tissues, can influence carbon and nitrogen cycling in soils.

Tannins can be broadly classified into condensed tannins (CTs) and hydrolysable tannins (HTs; Kraus et al., 2003a), with gymnosperms and monocots producing primarily CTs, and dicots able to produce either HTs or CTs alone or as mixtures (Bate-Smith, 1977; Haslam, 1988; Kraus et al., 2003a; Triebwasser et al., 2012). Condensed tannins, also known as proanthocyanidins, are polymers of flavan-3-ols such as catechin and gallocatechin (Xie and Dixon, 2005). Hydrolysable tannins are composed of gallic acid units linked via an ester linkage to a glucose functional group, and they can be further classified as gallotannins and ellagitannis by the respective presence or absence of C–C linked galloyl groups (Hartzfeld et al., 2002). In plants, tannin production is genetically (Scioneaux et al., 2011), as well as environmentally, controlled (Tharayil et al., 2011). Environmental conditions that affect the production of tannins include photoperiod, soil pH, moisture and nutrient availability, herbivory, and atmospheric CO_2_ and O_3_ (Herms and Mattson, 1992; Bussotti et al., 1998; Kraus et al., 2003a; Cohen and Kennedy, 2010; Jaakola and Hohtola, 2010; Lindroth, 2010; Malisch et al., 2016). Along with their total quantity, the biological reactivity of tannins is closely regulated by the chemistry of the polymer, *viz*. identity of monomers, substitution pattern of the phenolic ring, and the degree of polymerization (Kraus et al., 2003b). Although tannin quantities have been shown to respond to environmental changes, little is known about the influence of climate on the chemical composition of tannins. Further, less is known about the changes in the composition of tannins between green and senesced tissues, which are primarily influenced by the overall resorption metabolism during tissue senescence.

We investigated the influence of warming and altered precipitation on tannin production in *Quercus rubra*, an important species in many forested ecosystems of eastern North America (Prasad et al., 2007-ongoing). Specifically, we sought to determine the interactive effects of temperature and precipitation on (1) the content and molecular composition of tannins, (2) the association of tannins within different tissue fractions (operationally defined as extractable and non-extractable tannins) and (3) whether climatic effects on content and chemical composition of tannins differ between green and senescent leaves. We hypothesized that the climate stresses we imposed (drought, increased temperature) would induce the production of tannins that are quantitatively and compositionally different, and that the climate-induced changes in composition of tannins in green leaves would also influence the content of tannins that are retained in the senescent leaves.

## Methods and materials

### Site description

Samples were collected from the Boston-Area Climate Experiment (BACE), located in Waltham, Massachusetts, USA. The experiment is located in an old-field ecosystem, and the soil is a Mesic Typic Dystrudept (Haven series) with loamy topsoil (0–30 cm; sand:silt:clay ratios of 45:46:9) over a gravelly sandy loam subsoil. The BACE has four temperature treatments and three precipitation treatments and is divided into three replicate blocks with 12 plots (2 × 2 m, with 1 m spacing between plots) within each block. Lateral movement of water is prevented by lining each plot with polyethylene sheets to a depth of 0.6 m. Each block has ambient (i.e., control, unmanipulated), wet (150% of ambient rainfall during the growing season) and dry (50% of ambient rainfall year-round) precipitation zones, which are administered through a passive removal and active redistribution system mounted above the plots on otherwise open greenhouse frames. *Dry* plots are located under portions of the greenhouse frames that are covered in evenly spaced, clear, six-inch polycarbonate slats (15 cm slats arranged 15 cm apart) that exclude 50% of incoming precipitation. Frames over the *Ambient* and *Wet* treatments are covered in deer netting that provides similar shading, reducing photosynthetically active radiation by ~6% but letting all precipitation in. The precipitation collected from the dry plots was immediately reapplied to the *Wet* plots via electric pumps and an overhead sprinkler system (Hoeppner and Dukes, 2012). Within each precipitation zone are plots designated for one of four levels of warming: no warming, low (+c. 1.0°C), medium (+c. 2.7°C) and high (+c. 4.0°C) (Tharayil et al., 2011; Hoeppner and Dukes, 2012; Suseela and Dukes, 2013). The warming treatments were applied, starting in 2008, with infrared heaters that were mounted 1 m above each corner of the plot. Temperature at the middle of the plots was measured every 10 s. The difference in temperature between the plant canopies of the warmest and ambient plots within each group of four plots was used to achieve feedback control (Hoeppner and Dukes, 2012). Three bare-root plants (30–45 cm) of different tree species such as *Q. rubra, Betula lenta, Ulmus americanus*, and *Betula populifolia* were planted along the margins of each plot during April 2012. For this study, mature, non-shaded, leaves were collected from three *Q. rubra* trees per plot in 2013 during the first week of September (green leaves) and in the second week of October (freshly senesced leaves). Leaves of similar size were collected from 2 to 3 apical whorls per tree during each harvest, resulting in at least 12 leaves per plot per harvest. Leaves from trees within a plot were pooled together to obtain a composite sample. Leaves were flash frozen between slabs of dry ice immediately after harvest, and the samples were maintained below −20°C during all analyses. The *Q. rubra* leaves used for this study were collected from the unwarmed (*No Warm*) and high temperature (*Warm*) treatments exposed to *Dry, Ambient*, and *Wet* precipitation treatments. Three treatment replicates were maintained for each treatment in all analyses.

### Extraction of tissues

Leaf petioles were removed, and frozen leaf samples were finely ground in dry ice using mortar and pestle. Approximately 100 mg of the ground tissue was extracted twice with 10 ml of 100% MeOH. The methanol extracted tissues were further extracted with 10 ml of 75% acetone, three more times. During each extraction step the tissues were sonicated for 2 min, and then shaken for 2 h. The supernatant from the five extractions was pooled to obtain a composite extractable-fraction. The extracts were stored at −20°C and the residual litter (non-extractable) was washed with 2 ml of 80% methanol and dried overnight at 40°C.

### Condensed tannin quantity

The total content of condensed tannins (CTs) in the pooled extracts and in the litter residue was quantified using the acid-butanol assay modified from Porter et al. (1986). For the extractable tannins, subsamples (2 ml) of the pooled extract were dried down under N_2_ gas at 40°C before adding 6 ml of the butanol:HCl (95:5) reagent. For the analysis of the non-extractable tannins, approximately 20 mg of the residual litter was weighed into glass tubes and combined with 6 ml of the butanol:HCl reagent. Samples were then placed in a water bath at 90–95°C for an hour and then cooled on ice. The amount of depolymerized anthocyanidin in the samples was quantified spectrophotometrically by measuring the absorbance at 550 nm (Jasco V-550 UV/VIS spectrophotometer, Jasco, Analytical Instruments, Easton, MD, USA) with the amount of tannins quantified from a standard curve derived from purified tannins of *Q. rubra* collected from the same experimental plot as described in Tharayil et al. (2011). Since *Quercus* tannins are a mixture of CT and HTs, the weight percent of CTs in the purified tannins were determined during the depolymerizing of the purified tannins in the presence of excess phloroglucinol as described below. The CT concentration in the standard curve was adjusted accordingly to avoid the potential overestimation of tannins.

### Mean degree of polymerization and monomer identity of condensed tannin

Mass spectrometric analysis of intact polymeric tannins is challenging, especially when using electrospray as the ionization interface. This is because as the degree of polymerization increases the ionization efficiency of proanthocyanidins decreases (Karonen et al., 2006; Mouls et al., 2011). Also, stability of deprotonated proanthocyanidins and their fragments decreases with the increase in degree of polymerization of the molecule (Gu et al., 2003). Combined, this would result in an under sampling of the polymers with a higher chain-length, thus underestimating the degree of polymerization of proanthocyanidins in a sample. In order to further elucidate CT chemistry, subunit composition and mean degree of polymerization were determined by depolymerizing CTs in the presence of excess phloroglucinol (Kennedy and Jones, 2001; Karonen et al., 2006). Phloroglucinol reagent was prepared by dissolving 2.5 g phloroglucinol in 55 ml methanol containing 400 μl of concentrated HCl. The reagent was sparged with argon for ~30 min until the methanol volume was reduced to 50 ml. Two milliliters of the reagent were added to glass tubes containing ~150 mg of finely ground leaf tissues that were not subjected to prior solvent extraction. Samples in the tubes were then sparged with argon for another 2 min, immediately closed with Teflon-lined caps and incubated on a heating block at 50°C for 130 min. The tubes were then cooled on ice, centrifuged (1,000 *g*, 5 min), and 1.5 ml supernatant was transferred to centrifuge tubes. Saturated MgSO_4_ solution (~45 g in 100 ml water) was added to the supernatant and samples were cooled on ice again to facilitate the subsequent phase separation. For liquid-liquid extraction, 1.5 ml of ethyl ether was added and the tubes were shaken end to end on a rotatory shaker for 5 min. The tubes were centrifuged (1000 *g*, 5 min) and 40 μl of the top ethyl ether fraction was transferred to 100 μl glass inserts and completely dried under N_2_ gas. The samples were reconstituted in 50% methanol and analyzed using a liquid chromatograph coupled to a triple quadrupole mass spectrometer. Detailed chromatography and mass spectrometry parameters are given in supporting information.

Peak identities were determined based on authentic standards of catechin and gallocatechin as well as literature values reported for phloroglucinol adducts and procyanidin fragments (Table 1). Mass spectral patterns relative to un-adducted parent monomers were also compared. Concentrations of extractable catechin monomers were subtracted from the result of the phloroglucinol depolymerization assay. Relative mass responses (Kennedy and Jones, 2001) were used to normalize the difference in the ESI responses among the terminal units and phloroglucinol adducts. Procyanidin content was determined using the catechin and epicatechin monomers as well as fragments. Prodelphinidin content was defined as the gallocatechin/epi-gallocatechin pair (Table 1). Mean degree of polymerization of proanthocyanidins, was determined by dividing the sum of the normalized peak areas of extender units (phloroglucinol adducts of catechin, gallocatechin) by the sum of the peak areas of the terminal units (catechin and gallocatechin).

**Table 1:** **Identification and categorization of condensed tannin monomers**.

**Condensed Tannin Monomers**	**Categorization**	**[M–H]^−^**
(+)-Catechin	*Procyanidin*	289
(−)-Epicatechin	*Procyanidin*	289
(−)-Epigallocatechin	*Procyanidin*	305
(+)-Catechin with phloroglucinol adduct	*Procyanidin*	413
(−)-Epi-catechin with phloroglucinol adduct	*Procyanidin*	413
Epi-gallocatechin with phloroglucinol adduct	*Prodelphinidin*	429

*[M-H]^−^: deprotonated molecular mass*.

### Hydrolysable tannin analysis

For the quantification of hydrolysable tannins (HTs), methanolysis was carried out with a subsample of the methanol extract or 40 mg of methanol-extracted residual litter in methanolic H_2_SO_4_ at 85°C as described by Hartzfeld et al. (2002). The concentrations of methyl gallate were then quantified using high-pressure liquid chromatography (HPLC; see supporting information). Ellagic acid (ellagitannins), and methyl gallate (gallotannins), were identified and quantified in samples using authentic standards. Efficiency of methylation was monitored by measuring percent methylation of gallic acid standard under similar conditions as described above.

### Calculations and statistical analysis

The total concentration of tannins in the leaf tissue was calculated by summing CT and HT concentrations. A subsample of leaves collected at the same time was used for determining specific leaf area, which was similar between the green and freshly-senesced for all treatments meaning that trends observed on a mass basis would be similar if based on area (See Figure S1 in Suseela et al., 2015).

To test the main and interactive effects of warming and altered precipitation on CTs and HTs, a mixed model restricted maximum likelihood estimation (REML) with time of sampling (green or senesced) as repeated measures was used in SAS 9.2 (SAS Institute, Inc., Cary, North Carolina, USA). Degrees of freedom were calculated using the Kenward-Rogers method. Warming and precipitation treatments were fixed effects and blocks were treated as the random effects. Tukey's HSD multiple comparison test was used to identify differences among treatments. Significance was set at α = 0.05.

## Results

### Type of tannins

**Figure 1:**
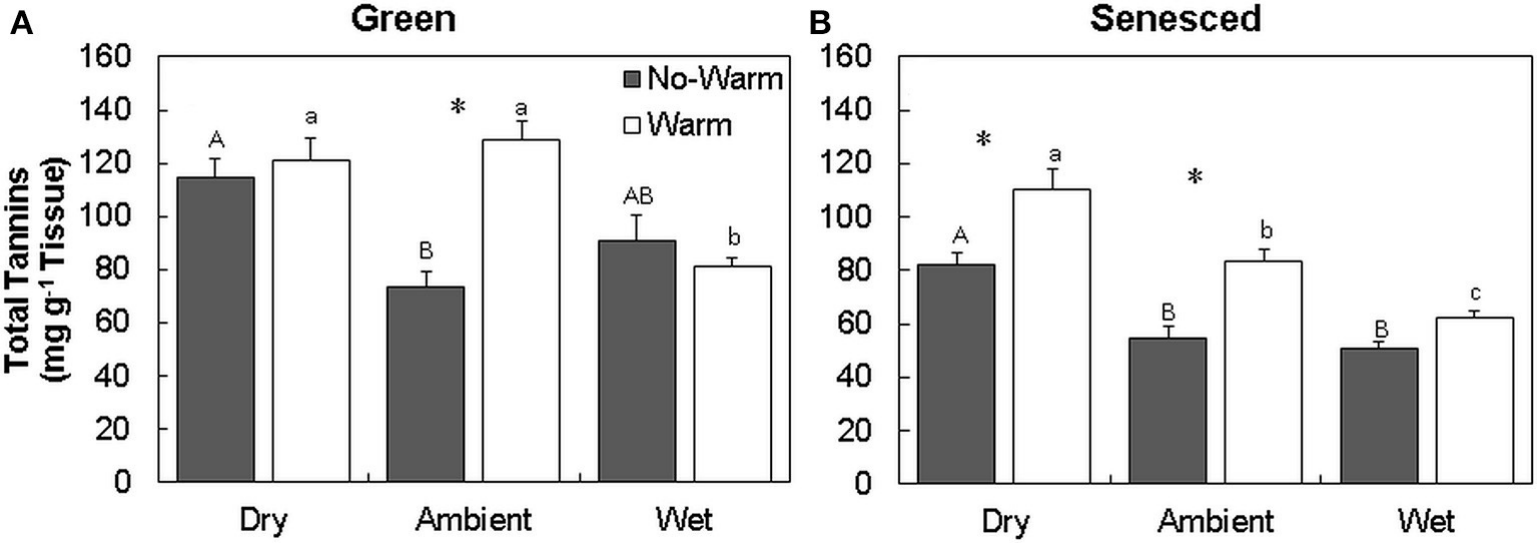
**Total average (±SE) tannin content for green (A)** and senesced **(B)** leaf tissue. Asterisks (^*^) above a set of columns indicate significant (*P* < 0.05) differences between *No Warm* and *Warm* treatments within each precipitation treatment. Uppercase letters indicate significant differences (*P* < 0.05) between precipitation treatments within the *No Warm* temperature regime. Lowercase letters indicate significant differences (*P* < 0.05) between precipitation treatments within the *Warm* temperature regime.

The total tannin concentration in green leaves varied with temperature and precipitation treatments (Figures 1A,B; Table S1). The green leaves formed in the Dry^*^Warm and Ambient^*^Warm treatment had the highest concentrations of total tannins (121.2 and 128.8 mg g^−1^ tissue, respectively; Figure 1A), while the Ambient^*^No Warm treatment had the lowest total tannin concentration (73.5 mg g^−1^ tissue; Figure 1A). Only the *Ambient* treatment exhibited an increase in green leaf tannin concentrations with an increase in temperature (*P* < 0.001). This pattern was also observed in senesced tissues, where the tannin concentration of senesced leaves also increased with higher temperature in both *Dry* (34% increase; *P* < 0.005) and *Ambient* (53% increase; *P* < 0.004) treatments (Figure 1B, Table S1). Within the *Warm* treatment, the senesced leaves in the drier treatments had more tannins (*P* < 0.05; Figure 1B). Dry^*^Warm had the highest concentration (110.5 mg g^−1^ tissue) and Wet^*^Warm had the lowest concentration (62.1 mg g^−1^ tissue) of total tannins (Figure 1B). The same trend was evident for senesced leaves exposed to *Dry, Ambient*, and *Wet* conditions in the *NoWarm* treatments. Decreases between the green and senesced tissue were observed in total tannins for the *Wet* treatments (*P* < 0.05) and the Ambient^*^No Warm treatment (*P* < 0.05).

When separating total tannins into CTs and HTs, CTs made up between 29 and 89% of the green leaf tissue and 53–88% of the senesced leaf tissue. In the green tissue of the Wet^*^No Warm treatment, HTs made up the larger percentage of total tannins (~69%) (Table 2). In green leaf tissue, warming did not alter HT concentrations in the *Dry, Ambient* (*P* = 0.06), or the *Wet* (*P* = 0.12) treatments (Table 2). The *Wet* precipitation treatment had the highest percentages of HTs in green leaf tissue for both the *No Warm* (69.1%) and the *Warm* (50.0%) treatments (Table 2). In senesced leaf tissue, warming increased (*P* < 0.001) the percent of HTs in the *Ambient* treatment, but decreased HTs (*P* < 0.001) in the *Wet* treatment (Table 2). In unwarmed plots, leaves in the *Ambient* treatment had the lowest percentage of HTs and those in the *Wet* treatment had the highest percentage (Table 2). Senesced leaf tissue had lower (*P* < 0.05) in HT percentages than green tissue in the all the *Wet* treatments and the Ambient^*^Warm treatment.

**Table 2:** **Percentages of hydrolysable tannins calculated from total tannins (±SE) for both green and senesced leaf tissue**.

**Treatments**		**Green**	**Senesced**
**Precipitation**	**Temperature**	**% HT**	**% HT**
Dry	No warm	30.7 ± 4.0^A^	21.2 ± 0.4^A^
Dry	Warm	24.4 ± 1.3^a^	20.6 ± 0.9^a^
Ambient	No warm	13.5 ± 1.4^A^	12.7 ± 0.6^*B^
Ambient	Warm	36.1 ± 0.9^ab^	26.0 ± 1.2^a^
Wet	No warm	69.1 ± 0.9^B^	42.2 ± 2.2^*C^
Wet	Warm	50.0 ± 2.0^b^	26.3 ± 1.2^a^

*Upper- and lower-case letters indicate significant (P < 0.05, Tukey's HSD) differences between precipitation treatments within the No Warm and Warm temperatures, respectively*.

Asterisk (^*^) indicates difference between No Warm and Warm within a specific precipitation treatment.

**Figure 2:**
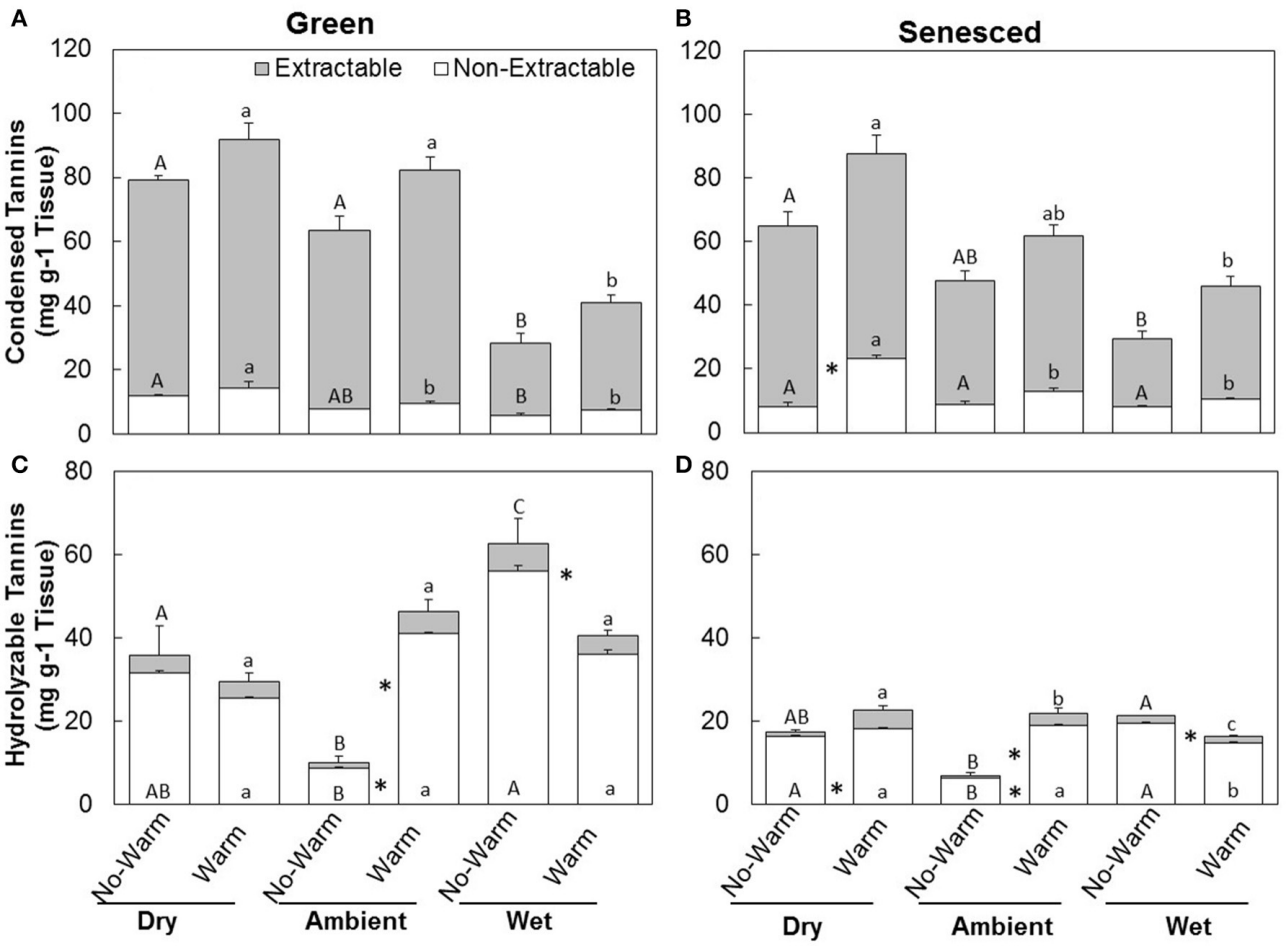
**Total average (±SE) condensed (A,B)** and hydrolysable **(C,D)** tannin content for the extractable and non-extractable portions of both green **(A,C)** and senesced **(B,D)** leaf tissue. Upper asterisks (^*^) indicate significant differences (*P* < 0.05) between the *No Warm* and *Warm* treatments within each precipitation treatment for extractable condensed tannins and lower asterisks indicate significant effects of warming within each precipitation treatment for non-extractable condensed tannins. Different upper case letters indicate significant differences (*P* < 0.05) between precipitation treatments within the *No Warm* temperature regime. Different lowercase letters indicate significant differences between precipitation treatments within the *Warm* treatment.

The responses of CT to climate treatments were similar to those of total tannins (Figures 2A,B). *Warm* treatments did not increase the total CTs in the green leaves in any of the precipitation treatments *Dry* (*P* = 0.41), *Ambient* (*P* = 0.14), or *Wet* (*P* = 0.42; Figure 2A). *Warm* treatments increased the total CTs in the senesced leaves for the *Dry* (*P* < 0.02), but not for the *Ambient* (*P* = 0.15) or the *Wet* (*P* = 0.082; Figure 2B). While total content of CTs did not change between the green and senesced leaf tissue, the content that CTs represented of the total proportion of tannins significantly (*P* < 0.05) increased for the Wet^*^No Warm and the Ambient^*^Warm and Wet^*^Warm.

Climatic treatments influenced the chemistry of CTs in leaf tissues (Table 3). The *Wet* treatment had CTs with longer subunit chains than in the *Dry* and *Ambient* treatments (Table 3). The proportion of epi/catechin to epi/gallocatechin or procyanidin (PC) to prodelphinidin (PD) differed only by precipitation treatment; warming did not change the percentage of CT monomers that were PD or PC. The *Ambient* treatment had the highest percentage of PDs (~27%) and *Wet* had the lowest, at <6.8%, with the *Dry* treatment intermediate (~10–14%; Table 3).

**Table 3:** **Percentage (±SE) of type of condensed tannins and average (±SE) chain length of condensed tannin polymers**.

**Treatments**		**PD %**	**Chain length (mean degree of polymerization)**
**Precipitation**	**Temperature**		
Dry	No warm	13.8 ± 1.5^a^	6.2 ± 0.5^b^
Dry	Warm	10.2 ± 1.7^a^	7.4 ± 0.6^b^
Ambient	No warm	27.7 ± 1.9^b^	6.1 ± 1.5^b^
Ambient	Warm	27.7 ± 1.3^b^	6.9 ± 0.5^b^
Wet	No warm	5.2 ± 0.3^c^	8.9 ± 0.8^a^
Wet	Warm	6.8 ± 0.5^c^	9.6 ± 1.4^a^

*Different letters indicate significant (P < 0.05, Tukey's HSD) differences between treatments*.

*PD refers to prodelphinidin, see Table 1*.

The total content of HTs in green tissues increased with warming under *Ambient* (*P* < 0.01) precipitation, but decreased with warming in the *Wet* treatment (*P* < 0.02; Figure 2C). In senesced tissue, HTs generally made up a lower percentage of the total tannin concentration than they did in green tissue (Figure 2D). Similar trends between *Warm* and *No Warm* treatments also appeared in the senesced tissue, except in the senesced leaf tissue the differences between *Warm* and *No Warm* were significant for all the precipitation treatments. Warming increased HTs in the *Dry* (*P* < 0.01) and the *Ambient* (*P* < 0.001) treatments, but decreased them in the *Wet* treatment (*P* < 0.005; Figure 2C). Total HT content significantly decreased from green to senesced leaf tissue in the Wet^*^No Warm treatment and all the *Warm* treatments.

The HTs can be further differentiated into two HT structural compounds in the *Q. rubra* leaves: ellagitannins and gallotannins (Tables 2, 4). Both green and senesced tissues generally had higher proportions of ellagitannins, although total contents were higher in the green tissue compared to the senesced tissue (Table 4). In the green tissue both gallotannins and ellagitannins displayed the same relationship, where the Ambient^*^No Warm treatment had the least (*P* < 0.05; Table 4). A similar relationship also existed in the senesced leaf tissue (Table 4). Over the course of the growing season, ellagitannin content decreased in green to senesced leaf tissue, but only in the *Wet* treatments (*No Warm, P* < 0.05; *Warm, P* < 0.001). Total gallotannin content also decreased (*P* < 0.05) between green and senesced leaf tissue in all the treatments except the Ambient^*^No Warm control.

**Table 4:** **Average concentrations, mg g^−1^ tissue, (±SE) of structural types of hydrolysable tannins for both green and senesced leaf tissue**.

**Treatments**		**Green**		**Senesced**	
**Precipitation**	**Temperature**	**Gallotannins**	**Ellagitannins**	**Gallotannins**	**Ellagitannins**
Dry	No warm	15.4 ± 2.0^A^	20.4 ± 4.8^A^	8.7 ± 0.5^A^	8.7 ± 0.1^*A^
Dry	Warm	12.6 ± 0.6^a^	18.8 ± 1.3^a^	10.0 ± 0.7^a^	12.6 ± 0.04^a^
Ambient	No warm	2.3 ± 0.2^*B^	7.8 ± 1.8^*B^	1.6 ± 0.2^*B^	5.3 ± 0.6^*B^
Ambient	Warm	20.3 ± 1.4^a^	26.2 ± 4.1^a^	9.9 ± 0.4^a^	11.8 ± 0.9^ab^
Wet	No warm	24.8 ± 2.2^*A^	37.9 ± 3.6^A^	10.9 ± 0.1^A^	10.3 ± 0.2^A^
Wet	Warm	15.2 ± 0.5^a^	25.4 ± 0.3^a^	7.3 ± 0.2^a^	9.0 ± 0.2^b^

*Upper- and lower-case letters indicate significant (P < 0.05, Tukey's HSD) differences between precipitation treatments within the No Warm and Warm temperatures, respectively*.

Asterisk (^*^) indicates difference between No Warm and Warm within a specific precipitation treatment.

### Tannin fractions

Total tannins, when separated into extractable and non-extractable fractions, showed similar patterns in both the green and senesced leaf tissue (Table 5). For the extractable fractions, the lowest concentration of tannins was in the *Wet* precipitation treatment, with no warming effect (Table 5). Warming increased the non-extractable fraction in green leaf tissue (*P* < 0.05) in the *Ambient* treatment (Table 5), and did not affect the non-extractable fraction in senesced tissue. In both the green and senesced leaf tissues, the lowest non-extractable tannin concentration existed in the Ambient^*^No Warm treatment (Table 5). Between seasons (green and senesced) there was a decrease (5.2%) in the Ambient^*^No Warm control and an increase (13.2%) in the Wet^*^Warm treatment in the percentage of tannins that were extractable.

**Table 5:** **Average concentrations, mg g^−1^ tissue, (±SE) of total extractable and non-extractable tannins for both green and senesced leaf tissue**.

**Treatments**		**Green**		**Senesced**	
**Precipitation**	**Temperature**	**Extractable**	**Non-extractable**	**Extractable**	**Non-extractable**
Dry	No warm	71.6 ± 2.1^A^	43.3 ± 7.6^A^	57.8 ± 4.7^A^	24.4 ± 1.0^A^
Dry	Warm	81.2 ± 5.1^a^	40.0 ± 3.6^a^	68.9 ± 5.8^a^	41.5 ± 1.6^a^
Ambient	No warm	57.2 ± 4.5^A^	16.4 ± 1.6^*B^	39.5 ± 3.2^AB^	15.0 ± 1.5^B^
Ambient	Warm	78.1 ± 4.0^a^	50.6 ± 3.0^a^	51.8 ± 3.2^ab^	31.9 ± 1.1^a^
Wet	No warm	29.2 ± 3.3^A^	61.8 ± 6.6^A^	23.0 ± 2.4^B^	27.7 ± 0.4^AB^
Wet	Warm	37.8 ± 1.6^a^	43.6 ± 1.4^a^	37.1 ± 3.0^b^	25.0 ± 0.1^a^

*Upper- and lower-case letters indicate significant (P < 0.05, Tukey's HSD) differences between precipitation treatments within the No Warm and Warm temperatures, respectively*.

Asterisk (^*^) indicates difference between No Warm and Warm within a specific precipitation treatment.

**Figure 3:**
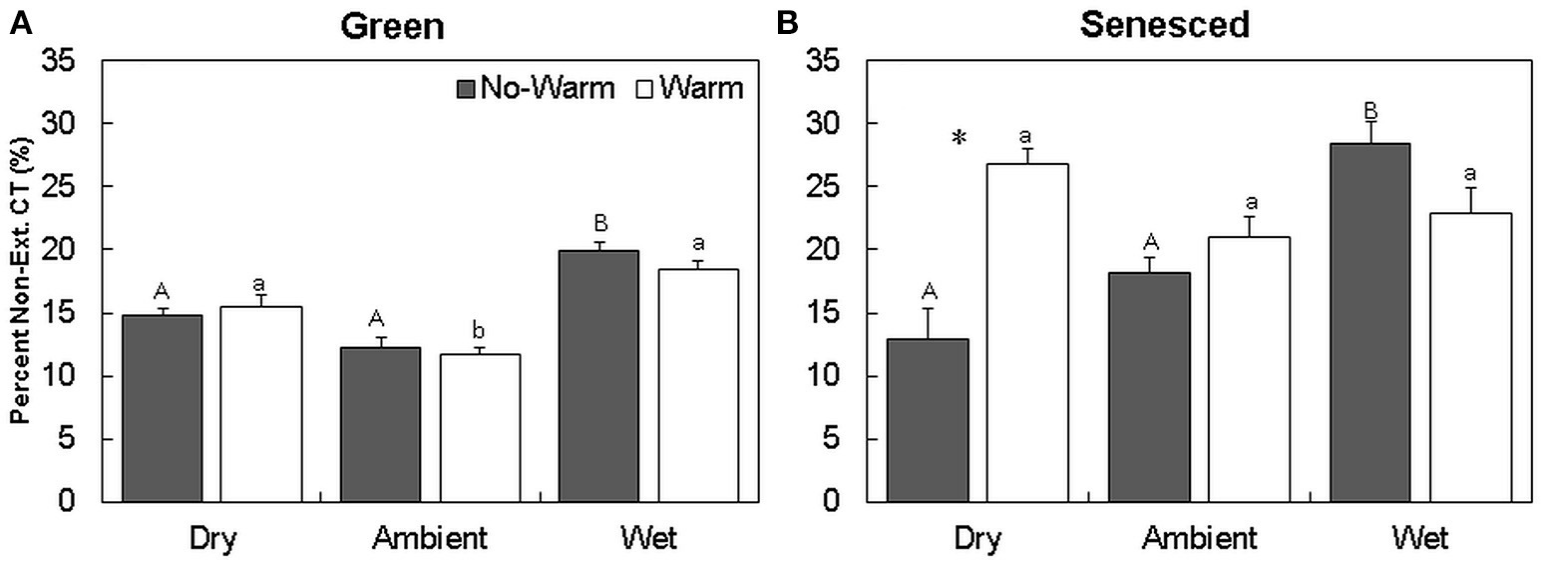
**Average (±SE) percent of non-extractable condensed tannins for green (A)** and senesced **(B)** leaf tissue. Asterisks (^*^) above a set of columns indicate significant (*P* < 0.05) differences between the *No Warm* and *Warm* treatments within each precipitation treatment. Different uppercase letters indicate significant differences between precipitation treatments within the *No Warm* temperature regime. Different lowercase letters indicate significant differences between precipitation treatments within the *Warm* temperature regime.

**Figure 4:**
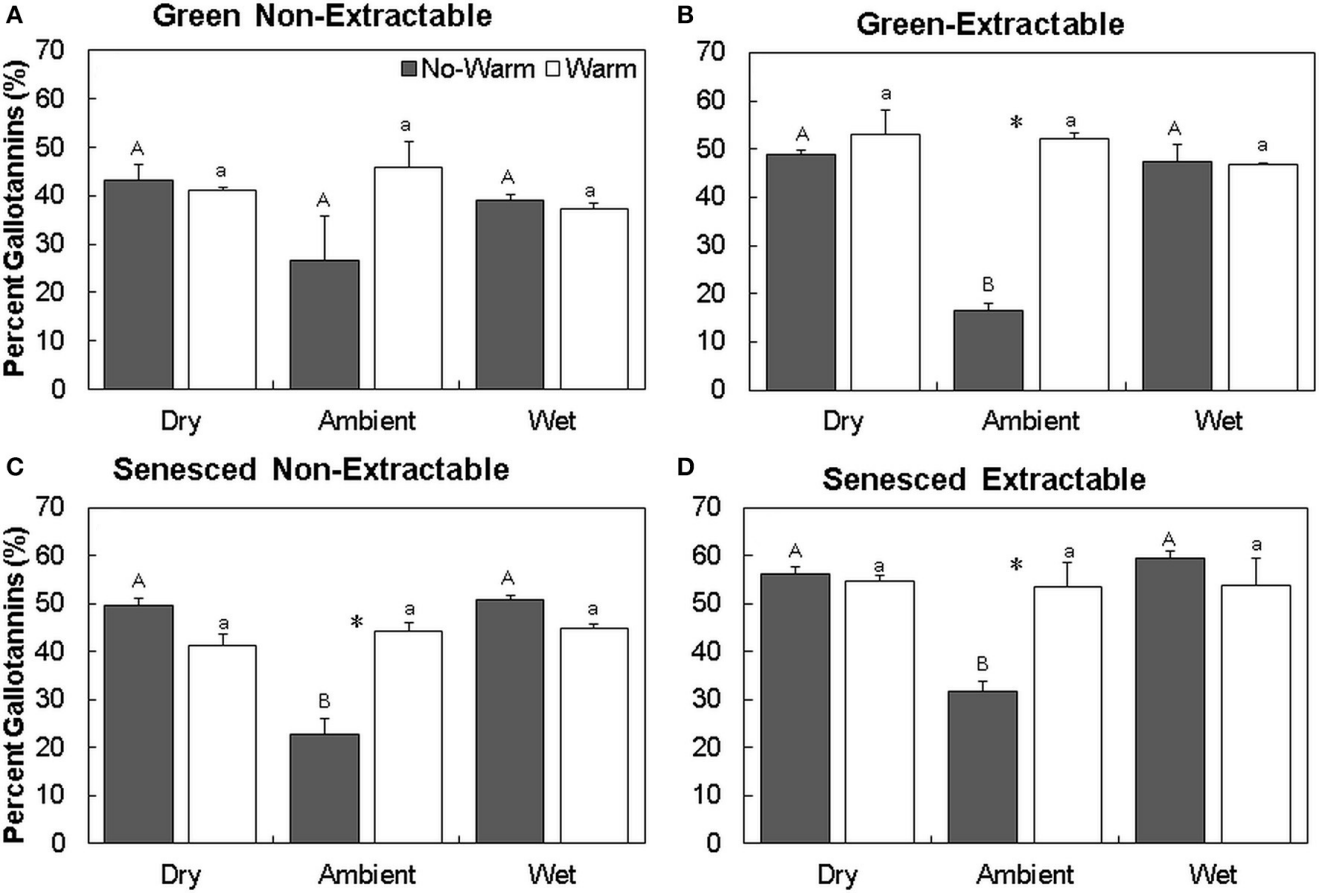
**Average (±SE) percent gallotannins in non-extractable and extractable hydrolysable tannins for both green (A,B)** and senesced **(C,D)** leaf tissue. Asterisks (^*^) above a set of columns indicate significant (*P* < 0.05) differences between *No Warm* and *Warm* treatments within each precipitation treatment. Uppercase letters indicate significant differences between precipitation treatments within the *No Warm* temperature regime. Lowercase letters indicate significant differences between precipitation treatments within the W*arm* temperature regime.

The majority of CTs were located in the extractable fraction (Figures 2A,B). Warming did not affect the total content of CTs in each fraction in the green leaf tissue (Figure 2). The percentage of non-extractable CTs in the green tissue was higher in the Wet^*^No Warm (*P* < 0.005), but not the Wet^*^Warm (*P* = 0.09; **Figure 4A**). The *Ambient* treatments had the lowest percentage of non-extractable CTs (*No Warm* = 12.2%, *Warm* = 11.7%; **Figure 4A**). In the senesced tissue, warming had an effect only in the *Dry* treatment, where it increased (*P* < 0.001) the content of CTs in the non-extractable fraction (Figure 2B). The percentage of non-extractable CTs tended to increase with warming only in the *Dry* treatment, and then only marginally (*P* < 0.1; Figure 3B). Within the *No Warm* temperature treatment, the *Wet* had the highest percentage (28.4%) of non-extractable CTs (Figure 3B). The percentage of non-extractable CTs significantly increased (by 8.5, 11.4, and 9.3%) from green leaves to senesced leaves for the Wet^*^No Warm, Dry^*^Warm, and Ambient^*^Warm, respectively.

The relative majority of HTs were located in the non-extractable fraction (Figures 2C,D) for both the green and senesced tissue. In green leaf tissue, warming increased the proportions of HTs in both the extractable (*P* < 0.03) and non-extractable (*P* < 0.005) fractions of the *Ambient* treatment (Figure 2C). Warming also decreased (*P* < 0.03) HTs in the non-extractable fraction in the *Wet* treatment (Figure 2C). Similar patterns occurred in the senesced tissue as well (Figure 2D), where HTs also increased with warming in the *Dry* treatment (Figure 2D).

For individual HTs, the percent of gallotannins increased the most in the *Ambient* treatment, with Ambient^*^No Warm having the lowest percentages of gallotannins (Figure 4). In the *Ambient* treatment, warming increased gallotannins in the extractable fraction in both the green and senesced leaf tissue (Figures 4B,D). In senesced tissue, warming in the *Ambient* treatment also increased the gallotannin percentage of the non-extractable fraction (this pattern did not occur in the green leaf tissue; *P* = 0.09, Figures 4A,C). In the *Wet* treatment, senesced leaves had more gallotannins (11.8%, *No Warm*; 7.7% *Warm*) in the non-extractable fraction than green leaf tissue.

## Discussion

Climatic stresses that disrupt cellular functioning in plants initiate readjustments in metabolic pathways, which in turn facilitate the acclimation of plants to their new environment. The resulting reprogramming of important metabolic pathways, including the phenylpropanoid pathway, citric acid cycle, glycolysis, and urea cycle, results in the upregulation of several metabolite classes such as amino acids, phenolic acids, sugars, organic acids, sugar alcohols, polyamines, and polyols (Bohnert et al., 1995; Peñuelas et al., 2013; Suseela et al., 2015). Drought and warmer temperatures have been shown to influence many different morphological (yield, growth) and physiological (rate of photosynthesis, stomatal conductance, leaf pigmentation, water potential, protein concentrations, etc.) responses in plants (Benjamin and Nielsen, 2006; Rennenberg et al., 2006; Praba et al., 2009; Anjum et al., 2011). However, the influence of environmental stressors on the content and composition of polymeric defense compounds across green and senesced plant tissues is less well-known.

In this study, climate-induced changes in leaf tannin composition influenced not only the quantity of tannins, but also the proportion of HTs to CTs, monomer composition and mean degree of polymerization of CTs, composition of HTs, the association of tannins within the cells (extractable and non-extractable), and the composition of the tannins remaining in the senescent leaves. In general, plants growing in favorable conditions (Ambient^*^No Warm, Wet) produced less tannins per unit leaf mass than those growing in more stressful conditions (Dry, Ambient^*^Warm). Increase in tissue content of tannins with increasing environmental stress has been frequently reported (Bussotti et al., 1998; Cohen and Kennedy, 2010). Reallocation of nutrients in plants based on the favorability of growth and nutrient conditions is expected under two major hypotheses: the carbon/nutrient balance and the growth/differentiation balance (Herms and Mattson, 1992). The increase in tannin production under Dry^*^Warm conditions could be a defense strategy in plants to protect the resources that they have already acquired (Herms and Mattson, 1992; Wright et al., 2010; Massad et al., 2014). At the same time, in the *Wet* treatments, because growth conditions were presumably closer to optimal, the decrease in tannin production could be a result of the plant preferentially allocating C for growth instead of defense (Bryant et al., 1983; Tuomi, 1992). Also, a greater concentration of tannins binding with cellulose and lignin matrices (Zucker, 1983; Bussotti et al., 1998) could potentially create a physical impedance to cell expansion. A similar allocation pattern of photosynthates for growth, rather than for defense compounds, has been previously reported in other tree species (Donaldson et al., 2006), where the plant growth was negatively correlated with phenolics and CTs. In a previous study conducted at BACE, the tannin content in senesced leaves of *Acer rubrum* was greater in *Dry* than in *Ambient* treatments, while the senesced leaves from *Wet* and the *Ambient* treatments had similar tannin contents (Tharayil et al., 2011). This contrasting pattern could be attributed to a difference in the physiological responses of the two species to stress, and also to differences in precipitation patterns between the two growing seasons. In the 2009 growing season (April to October; Tharayil et al., 2011), 720 mm of rain fell, as opposed to 606 mm during the 2013 growing season. Further, only half as much rain fell in the last 4 months of the 2013 growing season (July to October) as fell during that period in 2009 (210 mm and 428 mm, respectively). The larger amount of precipitation received by *A. rubrum* during the 2009 growing season would have led to a similar physiological water status between *Ambient* and *Wet* treatments, which could have resulted in similar tannin content between these two precipitation treatments (Tharayil et al., 2011). In the present study, the scarcer and less evenly distributed precipitation of 2013 likely exposed *Q. rubra* in *Ambient* to a greater physiological water stress, which could explain the observed similarity in tannin content between *Ambient* and *Dry* treatments.

Along with the total quantity of tannins, climate influenced both the monomer composition and mean degree of polymerization of tannins, the two parameters that are seldom investigated despite their regulatory influence on the potential biological reactivity of tannins (Zucker, 1983; Kraus et al., 2003b; Tharayil et al., 2011; Triebwasser et al., 2012). Reactivity of CT is a function of the hydroxylation pattern of the B-ring, and tannins with trihydroxy B-rings (PD) are more reactive than those with dihydroxy B-ring (PC). Both *Wet* and *Dry* treatments, despite their contrasting influences on total content of CTs, reduced the relative proportion of PD units. Compared to PCs, the PD units are formed only during later parts of cell development (Stafford et al., 1989). Thus, the lower accumulation of the PDs in wet climates could indicate a metabolic modification in CT biosynthesis. Under the metabolic stress in *Dry* conditions the triphenolic vicinal hydroxyls predispose PDs to oxidation and polymerization reactions (Close and Mcarthur, 2002; Aron and Kennedy, 2008) resulting in a lower proportion of PDs. This could partly explain the lower proportion of PDs in *Dry* treatments despite a higher production of CTs. Compared to *Ambient* and *Dry* treatments, the proportion of PD units and the mean degree of polymerization were higher under *Wet* treatments. The biological reactivity of tannins is a function of their degree of polymerization, where tannins with greater chain length have a higher capacity to complex with and precipitate proteins. Thus, though the leaves that were formed under ambient growing conditions and *Wet* treatments had a lower tannin quantity, these tannins would have a greater capacity to defend herbivory due to their higher biological reactivity contributed by a greater polymerization and higher proportion of PD units. Environmental conditions have been shown to influence the degree of polymerization of tannins (Cohen and Kennedy, 2010). A lower polymerization of tannins exposed to non-optimal growing conditions has been reported before (Tharayil et al., 2011) in senesced leaves of *Acer rubrum* from the same site. In *Vitis vinifera*, environmental stressors, especially moisture deficit, have been reported to increase the mean degree of polymerization of tannins (Kennedy et al., 2002; Cohen and Kennedy, 2010). In *Onobrychis viciifolia* the influence of drought on the mean degree of polymerization of CTs was dependent on otogeny of the plant (Malisch et al., 2016). These results indicate that the influence of environment on molecular composition of tannins could be regulated by the physiological stress perceived by the plant (as opposed to the magnitude of the treatment applied), the identity of the species, as well as their growth stage.

Distributions of tannins within leaves are highly species-specific (Kraus et al., 2003a), and within a species the cellular localization of tannins, herein operationally defined as solvent extractable and non-extractable tannins, can change based on environmental conditions (Gagné et al., 2006). In green leaves, CTs are usually stored in the cell vacuoles (Stafford, 1988; Bussotti et al., 1998; Marles et al., 2003), and thus are sequestered away from potential interactions with the structural matrix and plant metabolic compounds. During leaf maturation the tannins penetrate the cell walls (Bussotti et al., 1998), which may result in an increase in the fiber-bound proportion of tannins. These complexes can protect the cell wall or organelle against microbial attack, and can also delay the decomposition after senescence of the tissue (Zucker, 1983). Irrespective of the warming treatment, the leaves from the *Wet* treatment had a greater proportion of total CT that was non-extractable during the sequential solvent extraction. While we cannot be certain of the exact complexation of the tannins, some of them may be in association with cellulose and pectin (Le Bourvellec et al., 2009; Padayachee et al., 2012a,b; Jakobek, 2015) in the cell walls and middle lamellae. A high affinity of PCs binding to pectin has been observed (Le Bourvellec et al., 2009; Watrelot et al., 2013; Jakobek, 2015), meaning that tannins in the *Wet* treatment with the highest percentage of PCs and greater chain-length could have a greater complexation capacity with cell wall components. Alternatively, the greater chain-length and higher proportion of PC units would have resulted in lower extractability of tannins from the leaf tissues, resulting in the observed lower content of tannins in *Wet* treatments. Also, the *Wet* treatments, despite a lower content of CTs, have a higher percentage of HTs, which have a higher enzyme inhibition capacity compared to CTs (Triebwasser et al., 2012). Thus, overall, the leaves formed under *Wet* treatments would have a similar herbivore deterrence contributed by tannins, despite a lower content of CTs in the tissue.

Climate influenced the proportion of HTs and CTs. Across most treatments, the dominant type of tannin in *Q. rubra* was CT, with lower content of HTs. A similar, lower proportion of HT in red oak species has been reported before (Barbehenn et al., 2005). Compared to the Ambient^*^NoWarm control, all climate treatments had higher percentages of HTs, which may reflect the ease of maintenance, mobility, and greater responsiveness of HTs relative to CTs (Zucker, 1983; Shure et al., 1998). Because HTs are less associated with the structural matrix of the plants than CTs, they offer less resistance to normal cell growth and expansion, making them a preferable type of defense compound over CTs in environments that favor active plant growth (Zucker, 1983), as evidenced by the higher percentages of HTs in the *Wet* treatments. HTs are also thought to be metabolically cheaper; the metabolic cost for the formation of proanthocyanidin (monomers of CT) is 0.395 ATP equivalent g^−1^, where as that of HTs is 0.27 equivalent g^−1^ (Lewis and Yamamoto, 1989).

Both gallotannins and ellagitannins also exhibit antioxidant properties (Barbehenn et al., 2006), and thus may play a role in the inhibition of higher radical formation in leaves, especially under climatic stress. Ellagitannins can also be highly variable in structure (Zucker, 1983). This could have specific relevance inhibiting digestive enzymes in herbivore digestive tracts (Zucker, 1983), which would be an effective defense mechanism against herbivory for the plant. In most climates in this study, the green leaves exhibited higher content of ellagitannins, however, the Ambient^*^No Warm control had the highest percentage of ellagitannins compared to all other treatments, suggesting that the alteration of climate in any way can significantly alter the proportion of certain types of tannins present, either by production rates or utilization of certain hydrolysable tannin structural components, such as the glucose molecule (Zucker, 1983).

During tissue senescence, following the disruption of membrane-bound vesicles, CTs can form insoluble complexes with proteins and cell wall carbohydrates (Kraus et al., 2003a; Marles et al., 2003), which could contribute to an increase in the percent of non-extractable CTs in the senesced tissue. Differences in the content of tannins in green leaves compared to senesced leaves observed in this study may be the result of different overall resorption strategies of the plants. Considering their protein complexation capacity, polymeric nature, and the C-C linkages, the CT would less amenable to enzyme mediated catalysis and subsequent resorption. Due to their lower complexity and the presence of more labile ester bonds, the gallotannins would be more amenable to resorption during tissue senescence than CTs. Compared to the green tissues, the greater mobilization of HTs during senescence is reflected in the lower proportion of HTs in the senesced tissues across the treatments. The CT content of the *Wet* treatments were similar in both green and senesced tissues, which could be primarily attributed to the greater degree of polymerization and a greater PC content of tannins produced in this treatment, which would have resulted in a greater association of these tannins with the cell wall.

Tannins can complex with carbohydrates and proteins and this complexation can persist after the leaf senesces, potentially delaying decomposition. With the increase in CTs and non-extractable CTs for some of the treatments, this might mean that those leaves would break down more slowly, lose less material to leaching, and possibly provide more sustained nutrient input into the soil. The higher tannin content of foliage observed under less favorable growing conditions could be partly countered by the changes in allocation patterns at the tree level, since trees exposed to climatic stress often produce less leaf biomass. However, the reduced carbon input through litterfall in such ecosystems, coupled with higher content of phenolic compounds in these tissues, might impose a greater constraint on nutrient cycling in these soils. Extractable phenolics and tannins are lost rapidly from the leaves, suggesting that leaching is a primary route for loss of tannins from leaves (Benner et al., 1988; Schofield et al., 1998; Hernes et al., 2001), and lower molecular weight tannins are lost more rapidly than higher molecular weight tannins (Schofield et al., 1998). Thus, the *Wet* treatments with their higher length CTs could better retain some of the tannin-related defense capabilities which could interfere with decomposition processes. However, the overall herbivory and decomposition processes are influenced not only by the content of anti-nutrients in the tissues, but also by their overall nutrient content (Suseela et al., 2013; Almuzini et al., 2017), which in turn is also regulated by environmental stressors.

Overall, our study elucidates multiple factors that regulate the production and seasonal change of tannins in *Q. rubra*. In agreement with the nutrient-balance hypothesis, the tannin concentrations in tissues formed under favorable climates were lower, but the tannins produced were more complex and potentially more protective due to higher chain lengths of CTs and greater proportions of HTs. When exposed to climatic stress, *Q. rubra* responded by producing a greater quantity of tannins that were of shorter chain length. The differential influence of the environment on the production dynamics of plant metabolites is important in the context of global changes and carbon and nitrogen cycle feedbacks. Although over longer time scales climate modifies ecosystem processes through shifts in species composition, over shorter time scales the physiological adaptations of plants to changing climate could influence soil C and nutrient cycling through changes in chemical composition of biomass. These changes in tannin content and composition may alter forest dynamics, not only by influencing decomposition and nutrient cycling dynamics, but also by regulating herbivore dynamics.

## Author contributions

ST and NT contributed to the conceptualization of the project and design of the experiment, JD provided the experimental framework, ST collected and analyzed the data, CP and NT guided the analyses, ST drafted the article, all authors contributed to the critical revision of the article.

### Conflict of interest statement

The authors declare that the research was conducted in the absence of any commercial or financial relationships that could be construed as a potential conflict of interest.
