# Supplementary material for: Climate Influences the Content and Chemical Composition of Foliar Tannins in Green and Senesced Tissues of Quercus rubra
Source: Front Plant Sci. 2017 May 16;8:423. doi: 10.3389/fpls.2017.00423 (PMC5432568; doi:10.3389/fpls.2017.00423)
Supplement: Supplementary file 1 [file DataSheet1.pdf]

Climate influences the content and chemical composition of foliar tannins in green and senesced tissues of *Quercus rubra*

Sara M. Top, Caroline M. Preston, Jeffrey S. Dukes and Nishanth Tharayil

Sara M. Top (saramarietop@gmail.com)

Nishanth Tharayil (ntharay@clemson.edu)

Supplementary Material

Fig. S1. Specific leaf area of green and freshly senesced leaves of *Quercus rubra* exposed to six different climatic treatments. (A0, ambient precipitation+ no warming; AH, ambient precipitation+ high warming; W0, wet precipitation+ no warming; WH, wet precipitation+ high warming; D0, dry precipitation + no warming; DH, dry precipitation

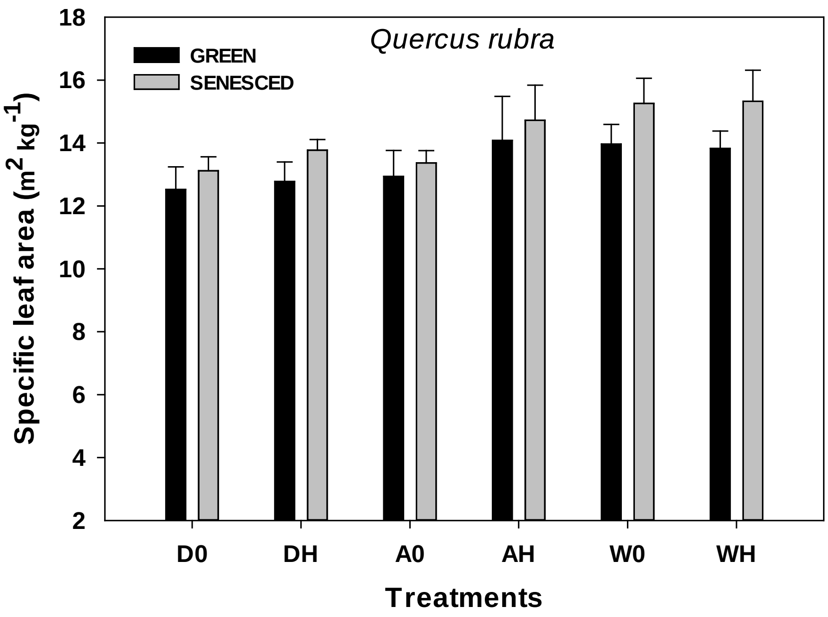

Condensed tannin analysis using liquid chromatography- mass spectrometry.

Analysis was done using a Shimadzu Ultra-Fast Liquid Chromatograph, equipped with a degasser and auto sampler connected in tandem to triple quadrupole mass spectrometer through an electrospray ionization interface (UFLC-ESI-MS/MS; Shimadzu 8030). Separations of depolymerized tannins were performed on a Kinetix XB-C18 column (100 mm x 4.16 mm ID; Phenomenex, Torrance, CA, USA). The mobile phase consisted of 0.03% formic acid (Solvent A) and acetonitrile (Solvent B) and flow rate was maintained at 0.5 ml min^{-1}. The proportion of acetonitrile was increased linearly from 10% to 85% over a 25 minute run. The triple quadrupole was operated in a scan mode from 260-550 m/z both in positive and negative ionization mode. The MS parameters were: DL temperature maintained at 230°C, heat block at 400°C, capillary voltage at 22kV, nebulizing gas of nitrogen at 3 L min^{-1} and curtain (drying) gas nitrogen at a rate of 14 L min^{-1}.

Hydrolyzable tannin analysis

The amounts of methyl gallate and ellagic acid were then quantified using high pressure liquid chromatography (HPLC). All the samples were analyzed with a Shimadzu quaternary pump UFLC system equipped with an autosampler, inline degasser, and UV–visible diode array detector. Separations were performed on a Gemini C_{18} column (5 µm 110A^0; 250 mm × 4.6 mm I.D.; Phenomenex, Torrance, CA). The mobile phase consisted of methanol:acetonitrile: water (10 : 5 : 85, v/v) buffered at pH 2.2 with 0.5% H_3PO_4. This gave a minimum peak resolution (R_s) of 4. The limit of detection was defined as having a signal-to-noise (*S/N*) ratio of 10 and all values reported are based on the peak area at 272nm.

Table S1. Statistical output (P-values) from the mixed model restricted maximum likelihood estimation for total tannin concentrations among precipitation, temperature and the interaction for both green and senesced leaf tissue.

	Green Leaf Tissue				*Senesced Leaf Tissue*			
	DF_n	DF_d	F-value	P-value	DF_n	DF_d	F-value	P-value
Total Tannin								
Precipitation	2	12	10.42	**0.0024**	2	9.98	52.25	**<0.0001**
Temperature	1	12	9.30	**0.0101**	1	9.98	49.47	**0.0001**
Ppt. x Temp.	2	12	11.72	**0.0015**	2	9.98	3.09	0.0903
Hydrolysable Tannin								
Precipitation	2	12	20.53	**0.0001**	2	10	51.49	**<0.0001**
Temperature	1	12	0.71	0.4152	1	10	107.58	**<0.0001**
Ppt. x Temp.	2	12	30.50	**<0.0001**	2	10	137.60	**<0.0001**
Condensed Tannin								
Precipitation	2	6.02	71.23	**<0.0001**	2	9.99	55.28	**<0.0001**
Temperature	1	5.98	16.81	**0.0064**	1	9.99	35.38	**0.0001**
Ppt. x Temp.	2	5.98	0.32	0.7368	2	9.99	0.75	0.4960
	Specific Hydrolysable Tannins							
Ellagitannins								
Precipitation	2	11	13.31	**0.0011**	2	12	7.03	**0.0095**
Temperature	1	11.1	0.00	0.9589	1	12	33.65	**<0.0001**
Ppt. x Temp.	2	11	15.43	**0.0006**	2	12	21.28	**0.0001**
Gallotannins								
Precipitation	2	12	7.35	**0.0082**	2	12	4.53	**0.0342**
Temperature	1	12	0.03	0.8556	1	12	2.03	0.1800
Ppt. x Temp.	2	12	23.25	**<0.0001**	2	12	12.26	**0.0013**

Bolded numbers indicate significant effects ($P < 0.05$).
Ppt. refers to precipitation
DF_n: Numerator degrees of freedom
DF_d: Denominator degrees of freedom
